# Affective rating of audio and video clips using the EmojiGrid

**DOI:** 10.12688/f1000research.25088.2

**Published:** 2021-04-06

**Authors:** Alexander Toet, Jan B. F. van Erp

**Affiliations:** 1 Perceptual and Cognitive Systems, TNO, Soesterberg, 3769DE, The Netherlands; 2 Department of Experimental Psychology, Helmholtz Institute, Utrecht University, Utrecht, 3584 CS, The Netherlands; 3 Research Group Human Media Interaction, University of Twente, Enschede, 7522 NH, The Netherlands

**Keywords:** affective response, audio clips, video clips, EmojiGrid, valence, arousal

## Abstract

**Background:** In this study we measured the affective appraisal of sounds and video clips using a newly developed graphical self-report tool: the EmojiGrid. The EmojiGrid is a square grid, labeled with emoji that express different degrees of valence and arousal. Users rate the valence and arousal of a given stimulus by simply clicking on the grid.

**Methods:** In Experiment I, observers (N=150, 74 males, mean age=25.2±3.5) used the EmojiGrid to rate their affective appraisal of 77 validated sound clips from nine different semantic categories, covering a large area of the affective space. In Experiment II, observers (N=60, 32 males, mean age=24.5±3.3) used the EmojiGrid to rate their affective appraisal of 50 validated film fragments varying in positive and negative affect (20 positive, 20 negative, 10 neutral).

**Results:** The results of this study show that for both sound and video, the agreement between the mean ratings obtained with the EmojiGrid and those obtained with an alternative and validated affective rating tool in previous studies in the literature, is excellent for valence and good for arousal. Our results also show the typical universal U-shaped relation between mean valence and arousal that is commonly observed for affective sensory stimuli, both for sound and video.

**Conclusions:** We conclude that the EmojiGrid can be used as an affective self-report tool for the assessment of sound and video-evoked emotions.

## Introduction

In daily human life, visual and auditory input from our environment significantly determines our feelings, behavior and evaluations ( Fazio, 2001; Jaquet *et al.,* 2014; Turley & Milliman, 2000, for a review see: Schreuder *et al.,* 2016). The assessment of the affective response of users to the auditory and visual characteristics of for instance (built and natural) environments ( Anderson *et al.,* 1983; Huang *et al.,* 2014; Kuijsters *et al.,* 2015; Ma & Thompson, 2015; Medvedev *et al.,* 2015; Toet *et al.,* 2016; Watts & Pheasant, 2015) and their virtual representations ( Houtkamp & Junger, 2010; Houtkamp *et al.,* 2008; Rohrmann & Bishop, 2002; Toet *et al.,* 2013; Westerdahl *et al.,* 2006), multimedia content ( Baveye *et al.,* 2018; Soleymani *et al.,* 2015), human-computer interaction systems ( Fagerberg *et al.,* 2004; Hudlicka, 2003; Jaimes & Sebe, 2010; Peter & Herbon, 2006; Pfister *et al.,* 2011) and (serious) games ( Anolli *et al.,* 2010; Ekman & Lankoski, 2009; Garner *et al.,* 2010; Geslin *et al.,* 2016; Tsukamoto *et al.,* 2010; Wolfson & Case, 2000) is an essential part of their design and evaluation and requires efficient methods to assess whether the desired experiences are indeed achieved. A wide range of physiological, behavioral and cognitive measures is currently available to measure the affective response to sensorial stimuli, each with their own advantages and disadvantages (for a review see: Kaneko *et al.,* 2018a). The most practical and widely used instruments to measure affective responses are questionnaires and rating scales. However, their application is typically time-consuming and requires a significant amount of mental effort (people typically find it difficult to name their emotions, especially mixed or complex ones), which affects the experience itself ( Constantinou *et al.,* 2014; Lieberman, 2019; Lieberman *et al.,* 2011; Taylor *et al.,* 2003; Thomassin *et al.,* 2012; for a review see: Torre & Lieberman, 2018) and restricts repeated application. While verbal rating scales are typically more efficient than questionnaires, they also require mental effort since users are required to relate their affective state to verbal descriptions (labels). Graphical rating tools however allow users to intuitively project their feelings to figural elements that correspond to their current affective state.

Arousal and pleasantness (valence) are principal dimensions of affective responses to environmental stimuli ( Mehrabian & Russell, 1974). A popular graphical affective self-report tool is the Self-Assessment Mannikin (SAM) ( Bradley & Lang, 1994): a set of iconic humanoid figures representing different degrees of valence, arousal, and dominance. Users respond by selecting from each of the three scales the figure that best expresses their own feeling. The SAM has previously been used for the affective rating of video fragments (e.g., Bos *et al.,* 2013; Deng *et al.,* 2017; Detenber *et al.,* 2000; Detenber *et al.,* 1998; Ellard *et al.,* 2012; Ellis & Simons, 2005; Fernández *et al.,* 2012; Soleymani *et al.,* 2008) and auditory stimuli (e.g., Bergman *et al.,* 2009; Bradley & Lang, 2000; Lemaitre *et al.,* 2012; Morris & Boone, 1998; Redondo *et al.,* 2008; Vastfjall *et al.,* 2012). Although the SAM is validated and widely used, users often misunderstand the depicted emotions ( Hayashi *et al.,* 2016; Yusoff *et al.,* 2013): especially the arousal dimension (shown as an ‘explosion’ in the belly area) is often interpreted incorrectly ( Betella & Verschure, 2016; Broekens & Brinkman, 2013; Chen *et al.,* 2018; Toet *et al.,* 2018). The SAM also requires a successive assessment of the stimulus on each of its individual dimensions. To overcome these problems we developed an alternative intuitive graphical self-report tool to measure valence and arousal: the EmojiGrid ( Toet *et al.,* 2018). The EmojiGrid is a square grid (resembling the Affect Grid: Russell *et al.,* 1989), labeled with emoji that express various degrees of valence and arousal. Emoji are facial icons that can elicit the same range of neural ( Gantiva *et al.*, 2020) and emotional ( Moore *et al.,* 2013) responses as real human faces. In contrast to photographs, emoji are not associated with overgeneralization (the misattribution of emotions and traits to neutral human faces that merely bear a subtle structural resemblance to emotional expressions: Said *et al.,* 2009), or racial, cultural and sexual biases. Although some facial emoji can be poly-interpretable ( Miller *et al.,* 2016; Tigwell & Flatla, 2016) it has been found that emoji with similar facial expressions are typically attributed similar meanings ( Jaeger & Ares, 2017; Moore *et al.,* 2013) that are also to a large extent language independent ( Novak *et al.,* 2015). Emoji have a wide range of different applications, amongst others in psychological research ( Bai *et al.,* 2019). Emoji based rating tools are increasingly becoming popular tools as self-report instruments ( Kaye *et al.,* 2017) to measure for instance user and consumer experience (e.g. www.emojiscore.com). Since facial expressions can communicate a wide variety of both basic and complex emotions emoji-based self-report tools may also afford the measurement and expression of mixed (complex) emotions that are otherwise hard to verbalize ( Elder, 2018). However, while facial images and emoji are processed in a largely equivalent manner, suggesting that some non-verbal aspects of emoji are processed automatically, further research is required to establish whether they are also emotionally appraised on an implicit level ( Kaye *et al.,* 2021).

The EmojiGrid enables users to rate the valence and arousal of a given stimulus by simply clicking on the grid. It has been found that the use of emoji as scale anchors facilitates affective over cognitive responses ( Phan *et al.,* 2019). Previous studies on the assessment of affective responses to food images ( Toet *et al.,* 2018) and odorants ( Toet *et al.,* 2019) showed that the EmojiGrid is self-explaining: valence and arousal ratings did not depend on framing and verbal instructions ( Kaneko *et al.,* 2019; Toet *et al.,* 2018). The current study was performed to investigate the EmojiGrid for the affective appraisal of auditory and visual stimuli.

Sounds can induce a wide range of affective and physiological responses ( Bradley & Lang, 2000; Gomez & Danuser, 2004; Redondo *et al.,* 2008). Ecological sounds have a clear association with objects or events. However, music can also elicit emotional responses that are as vivid and intense as emotions that are elicited by real-world events ( Altenmüller *et al.,* 2002; Gabrielsson & Lindström, 2003; Krumhansl, 1997) and can activate brain regions associated with reward, motivation, pleasure and the mediation of dopaminergic levels ( Blood & Zatorre, 2001; Brown *et al.,* 2004; Menon & Levitin, 2005; Small *et al.,* 2001). Even abstract or highly simplified sounds can convey different emotions ( Mion *et al.,* 2010; Vastfjall *et al.,* 2012) and can elicit vivid affective mental images when they have some salient acoustic properties in common with the actual sounds. As a result, auditory perception is emotionally biased ( Tajadura-Jiménez *et al.,* 2010; Tajadura-Jiménez & Västfjäll, 2008). Video clips can also effectively evoke various affective and physiological responses ( Aguado *et al.,* 2018; Carvalho *et al.,* 2012; Rottenberg *et al.,* 2007; Schaefer *et al.,* 2010). While sounds and imagery individually elicit various affective responses that recruit similar brain structures ( Gerdes *et al.,* 2014), a wide range of non-linear interactions at multiple processing levels in the brain make that their combined effects are not a priori evident (e.g., Spreckelmeyer *et al.,* 2006; for a review see: Schreuder *et al.,* 2016). Several standardized and validated affective databases have been presented to enable a systematic investigation of sound ( Bradley & Lang, 1999; Yang *et al.,* 2018) and video ( Aguado *et al.,* 2018; Carvalho *et al.,* 2012; Hewig *et al.,* 2005; Schaefer *et al.,* 2010) elicited affective responses.

This study evaluates the EmojiGrid as a self-report tool for the affective appraisal of auditory and visual events. In two experiments, participants were presented with different sound and video clips, covering both a large part of the valence scale and a wide range of semantic categories. The video clips were stripped of their sound channel (silent) to avoid interaction effects. After perceiving each stimulus, participants reported their affective appraisal (valence and arousal) using the EmojiGrid. The sound samples ( Yang *et al.,* 2018) and video clips ( Aguado *et al.,* 2018) had been validated in previous studies in the literature using 9-point SAM affective rating scales. This enables an evaluation of the EmojiGrid by directly comparing the mean affective ratings obtained with it to those that were obtained with the SAM.

In this study we also investigate how the mean valence and arousal ratings for the different stimuli are related. Although the relation between valence and arousal for affective stimuli varies between individuals and cultures ( Kuppens *et al.,* 2017), it typically shows a quadratic (U-shaped) form across participants (i.e., at the group level): stimuli that are on average rated either high or low on valence are typically also rated as more arousing than stimuli that are on average rated near neutral on valence ( Kuppens *et al.,* 2013; Mattek *et al.,* 2017). For the valence and arousal ratings obtained with the EmojiGrid, we therefore also investigate to what extent a quadratic form describes their relation at the group level. 

## Methods

### Participants

English speaking participants from the UK were recruited via the Prolific database ( https://www.prolific.co/). Exclusion criteria were age (outside the range of 18–35 years old) and hearing or (color) vision deficiencies. No further attempts were made to eliminate any sampling bias.

We estimated the sample size required for this study with the “ ICC.Sample.Size” R-package, assuming an ICC of 0.70 (generally considered as ‘moderate’: Landis & Koch, 1977), and determined that sample sizes of 57 (Experiment 1) and 23 (Experiment 2) would yield a 95% confidence interval of sufficient precision (±0.07; Landis & Koch, 1977). Because the current experiment was run online and not in a well-controlled laboratory environment, we aimed to recruit about 2–3 times the minimum required number of participants.

This study was approved by the by TNO Ethics Committee (Application nr: 2019-012), and was conducted in accordance with the Helsinki Declaration of 1975, as revised in 2013 ( World Medical Association, 2013). Participants electronically signed an informed consent by clicking “ *I agree to participate in this study*”, affirming that they were at least 18 years old and voluntarily participated in the study. The participants received a small financial compensation for their participation.

### Measures

***Demographics.*** The participants in this study reported their nationality, gender and age.

**Figure 1.**
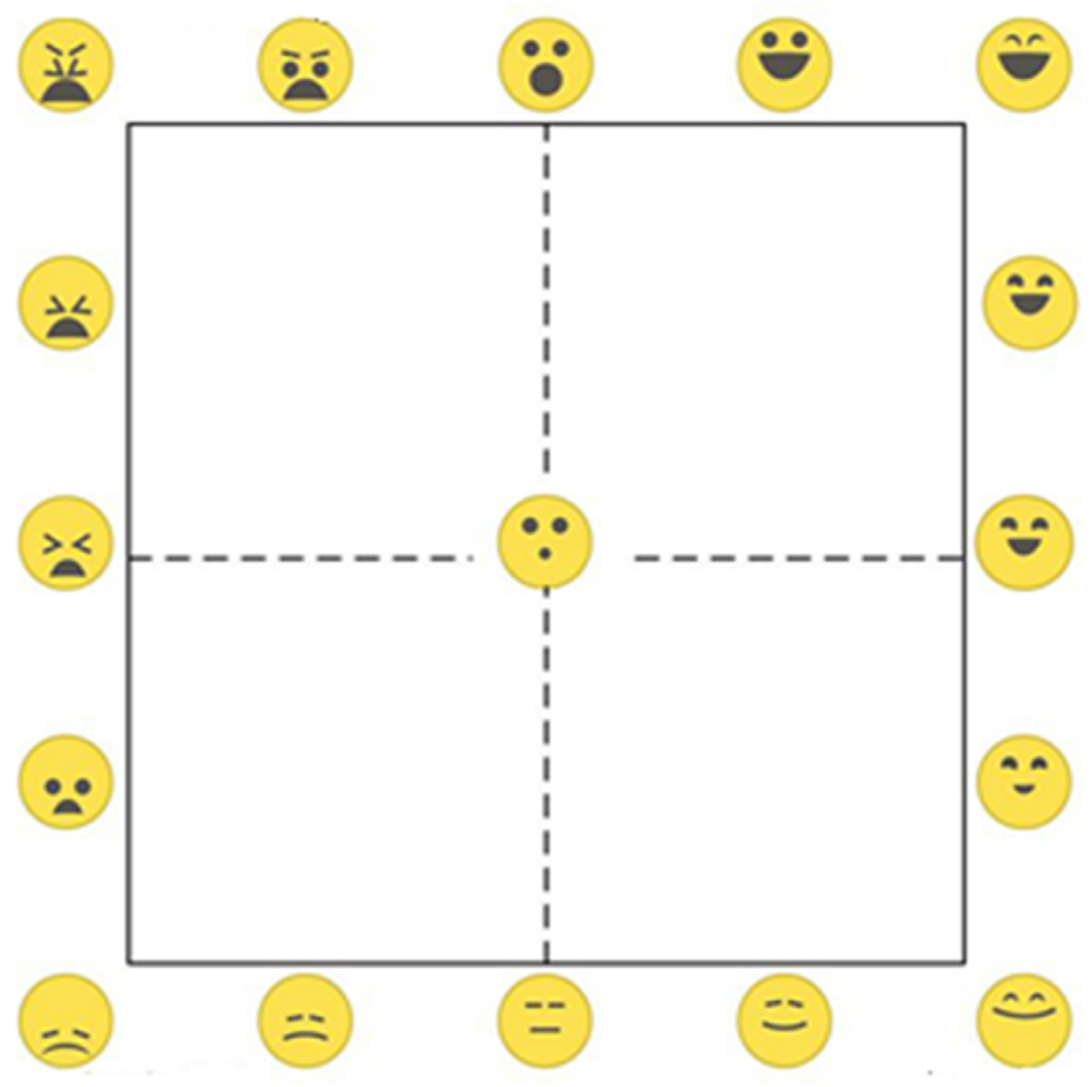
The EmojiGrid. The iconic facial expressions range from disliking (unpleasant) via neutral to liking (pleasant) along the horizontal (valence) axis, while their intensity increases along the vertical (arousal) axis. This figure has been reproduced with permission from Toet *et al.,* 2018.

***Valence and arousal: the EmojiGrid.*** The EmojiGrid is a square grid (similar to the Affect Grid: Russell *et al.,* 1989), labeled with emoji that express various degrees of valence and arousal ( Figure 1). Users rate their affective appraisal (i.e., the valence and arousal) of a given stimulus by pointing and clicking at the location on the grid that that best represents their impression. The EmojiGrid was originally developed and validated for the affective appraisal of food stimuli, since the SAM appeared to be frequently misunderstood in that context ( Toet *et al.,* 2018). It has since also been used and validated for the affective appraisal of odors ( Toet *et al.,* 2019).

### Procedure

Participants took part in two anonymous online surveys, created with the Gorilla experiment builder ( Anwyl-Irvine *et al.,* 2019). After thanking the participants for their interest, the surveys first gave a general introduction to the experiment. The instructions asked the participants to perform the survey on a computer or tablet (but not on a device with a small screen such as a smartphone) and to activate the full-screen mode of their browser. This served to maximize the resolution of the questionnaire and to prevent distractions by other programs running in the background. In Experiment I (sounds) the participants were asked to turn off any potentially disturbing sound sources in their room. Then the participants were informed that they would be presented with a given number of different stimuli (sounds in Experiment I and video clips in Experiment II) during the experiment and they were asked to rate their affective appraisal of each stimulus. The instructions also mentioned that it was important to respond seriously, while there would be no correct or incorrect answers. Participants could electronically sign an informed consent. By clicking “ *I agree to participate in this study* ”, they confirmed that they were at least 18 years old and that their participation was voluntary. The survey then continued with an assessment of the demographic variables (nationality, gender, age).

Next, the participants were familiarized with the EmojiGrid. First, it was explained how the tool could be used to rate valence and arousal for each stimulus. The instructions were: “ *To respond, first place the cursor inside the grid on a position that best represents how you feel about the stimulus, and then click the mouse button.*” Note that the dimensions of valence and arousal were not mentioned here. Then the participants performed two practice trials. In Experiment I, these practice trials also allowed the repeated playing of the sound stimulus. This was done to allow the participants to adjust the sound level of their computer system. The actual experiment started immediately after the practice trials. The stimuli were presented in random order. The participants rated each stimulus by clicking at the appropriate location on the EmojiGrid. The next stimulus appeared immediately after clicking. There were no time restrictions. On average, each experiment lasted about 15 minutes.

Experiment I: Sounds

This experiment served to validate the EmojiGrid as a rating tool for the affective appraisal of sound-evoked emotions. Thereto, participants rated valence and arousal for a selection of sounds from a validated sound database using the EmojiGrid. The results are compared with the corresponding SAM ratings provided for each sound in the database.

***Stimuli.*** The sound stimuli used in this experiment are 77 sound clips from the expanded version of the validated International Affective Digitized Sounds database (IADS-E, available upon request; Yang *et al.,* 2018). The sound clips were selected from 9 different semantic categories: scenarios (2), breaking sounds (8), daily routine sounds (8), electric sounds (8), people (8), sound effects (8), transport (8), animals (9), and music (10). For all sounds, Yang *et al.* (2018) provided normative ratings for valence and arousal, obtained with 9-point SAM scales and collected by at least 22 participants from a total pool of 207 young Japanese adults (103 males, 104 females, mean age 21.3 years, SD=2.4). The selection used in the current study was such that the mean affective (valence and arousal) ratings provided for stimuli in the same semantic category were maximally distributed over the two-dimensional affective space (ranging from very negative like a car horn, hurricane sounds or sounds of vomiting, via neutral like people walking up a stairs, to very positive music). As a result, the entire stimulus set is a representative cross-section of the IADS-E covering a large area of the affective space. All sound clips had a fixed duration of 6s. The exact composition of the stimulus set is provided in the Supplementary Material. Each participant rated all sound clips.

***Participants.*** A total of 150 participants (74 males, 76 females) participated in this experiment. All participants were UK nationals. Their mean age was 25.2 (SD= 3.5) years.

Experiment II: Video clips

This experiment served to validate the EmojiGrid as a self-report tool for the assessment of emotions evoke by (silent) video clips. Participants rated valence and arousal for a selection of video clips from a validated set of video fragments using the EmojiGrid. The results are compared with the corresponding SAM ratings for the video clips ( Aguado *et al.,* 2018).

***Stimuli.*** The stimuli comprised of a set of 50 film fragments with different affective content (20 positive ones like a coral reef with swimming fishes and jumping dolphins, 10 neutral ones like a man walking in the street or an elevator going down, and 20 negative ones like someone being attacked or a car accident scene). All video clips had a fixed duration of 10 s and were stripped of their soundtracks (for detailed information about the video clips and their availability see Aguado *et al.,* 2018). Aguado *et al.* (2018) obtained normative ratings for valence and arousal, collected by 38 young adults (19 males, 19 females, mean age 22.3 years, SD=2.2) using 9-point SAM scales. In the present study, each participant rated all video clips using the EmojiGrid.

***Participants.*** A total of 60 participants (32 males, 28 females) participated in this experiment. All participants were UK nationals. Their mean age was 24.5 (SD= 3.3) years.

### Data analysis

The response data (i.e., the horizontal or valence and vertical or arousal coordinates of the check marks on the EmojiGrid) were quantified as integers between 0 and 550 (the size of the square EmojiGrid in pixels), and then scaled between 1 and 9 for comparison with the results of Yang *et al.* (2018) obtained with a 9-point SAM scale (Experiment I), or between 0 and 8 for comparison with the results of Aguado *et al.* (2018), also obtained with a 9-point SAM scale (Experiment II).

All statistical analyses were performed with IBM SPSS Statistics 26 ( www.ibm.com) for Windows. The computation of the intraclass correlation coefficient (ICC) estimates with their associated 95% confidence intervals was based on a mean-rating (k = 3), consistency, 2-way mixed-effects model ( Koo & Li, 2016; Shrout & Fleiss, 1979). ICC values less than 0.5 indicate poor reliability, values between 0.5 and 0.75 suggest moderate reliability, values between 0.75 and 0.9 represent good reliability, while values greater than 0.9 indicate excellent reliability ( Koo & Li, 2016; Landis & Koch, 1977). For all other analyses a probability level of p < 0.05 was considered to be statistically significant.

MATLAB 2020a was used to further investigate the data. The mean valence and arousal responses were computed across all participants and for each of the stimuli. MATLAB’s Curve Fitting Toolbox (version 3.5.7) was used to compute least-squares fits to the data points. Adjusted R-squared values were calculated to quantify the agreement between the data and the curve fits.

## Results

### Experiment I

**Figure 2.**
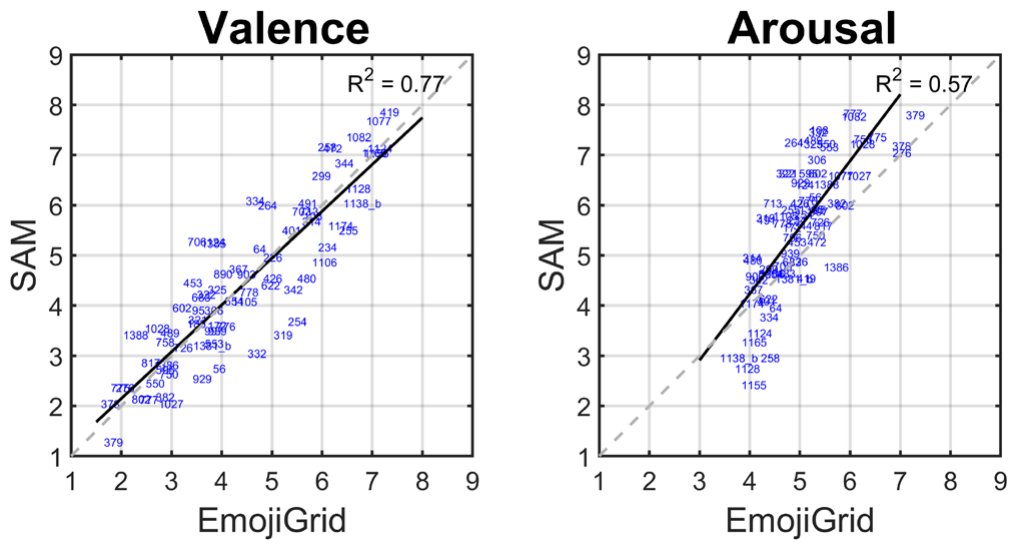
Relation between mean valence (left) and arousal (right) ratings obtained with the SAM and EmojiGrid for selected sounds from the IADS-E database. Labels correspond to the original identifiers of the stimuli ( Yang *et al.*, 2018). The line segments represent linear fits to the data points.

Figure 2 shows the correlation plots between the mean valence and arousal ratings for the 77 affective IADS-E sounds used in the current study, obtained with the EmojiGrid (this study) and with a 9-point SAM scale ( Yang *et al.* (2018). This figure illustrates the overall agreement between the affective ratings obtained with both self-assessment tools for affective sound stimuli.

The linear (two-tailed) Pearson correlation coefficients between the valence and arousal ratings obtained with the EmojiGrid (present study) and with the SAM ( Yang *et al.*, 2018) were, respectively, 0.881 and 0.760 (p<0.001). To further quantify the agreement between both rating tools we computed intraclass correlation coefficients (ICC) with their 95% confidence intervals for the mean valence and arousal ratings between both studies. The ICC value for valence is 0.936 [0.899–0.959] while the ICC for arousal is 0.793 [0.674–0.868], indicating both studies show an excellent agreement for valence and a good agreement for arousal (even though the current study was performed via the internet and therefore did not provide the amount of control over many experimental factors as one would have in a lab experiment).

**Figure 3.**
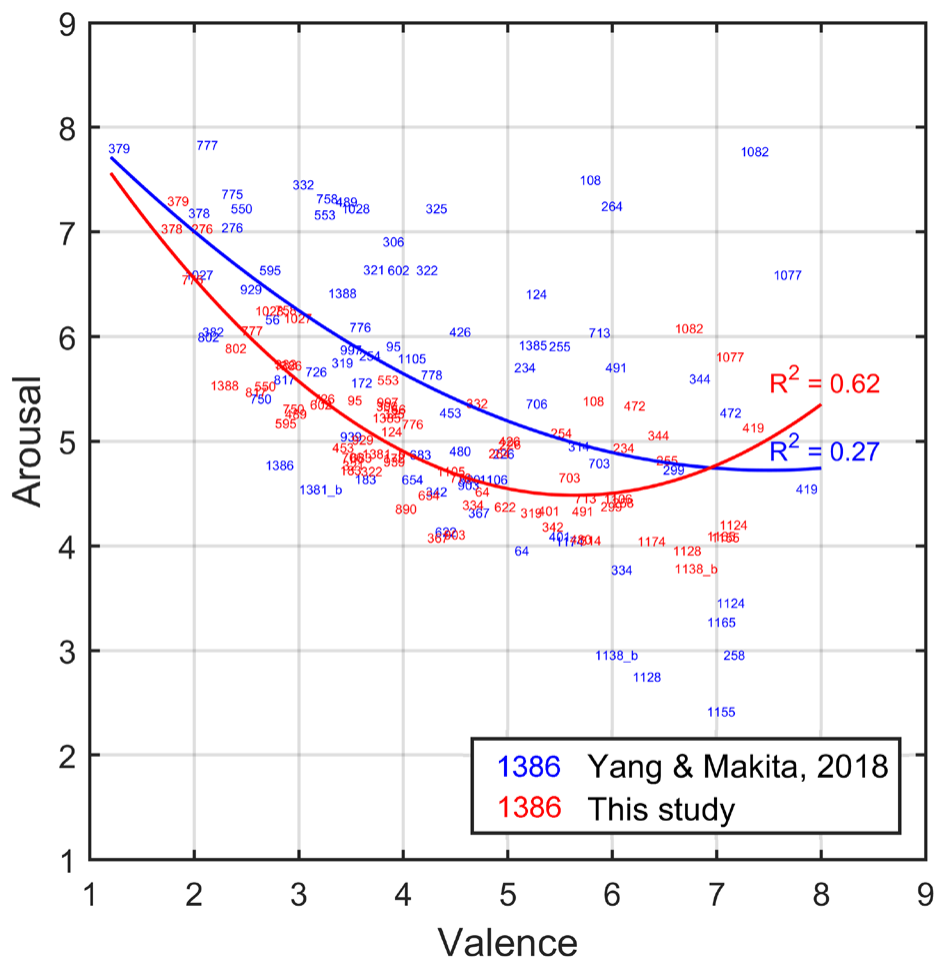
Relation between mean valence and arousal ratings for selected sounds from the IADS-E database. Labels correspond to the original identifiers of the stimuli ( Yang *et al.*, 2018). Blue labels represent data obtained with the SAM ( Yang *et al.*, 2018), while red labels represent data obtained with the EmojiGrid (this study). The curves represent quadratic fits to the corresponding data points.

Figure 3 shows the relation between the mean valence and arousal ratings for the 77 IADS-E sounds used as stimuli in the current study, measured both with the EmojiGrid (this study) and with a 9-point SAM scale ( Yang *et al.* (2018). The curves in this figure represent least-squares quadratic fits to the data points. The adjusted R-squared values are 0.62 for results obtained with the EmojiGrid and 0.22 for the SAM results. Hence, both methods yield a relation between mean valence and arousal ratings that can indeed be described by a quadratic (U-shaped) relation at the nomothetic (group) level.

### Experiment II

**Figure 4.**
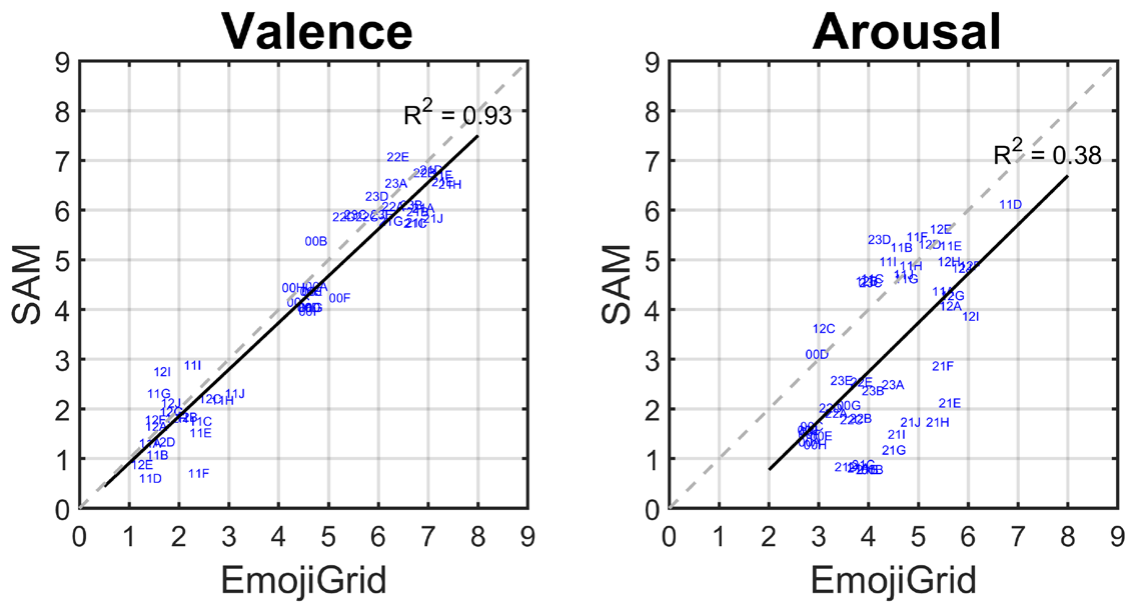
Relation between mean valence (left) and arousal (right) ratings obtained with the SAM and EmojiGrid for 50 affective video clips ( Aguado *et al.*, 2018). Labels correspond to the original identifiers of the stimuli ( Yang *et al.*, 2018). The line segments represent linear fits to the data points.

Figure 4 shows the correlation plots between the mean valence and arousal ratings for the 50 affective video clips used in the current study, obtained with the EmojiGrid (this study) and with a 9-point SAM scale ( Aguado *et al.*, 2018). This figure illustrates the overall agreement between the affective ratings obtained with both self-assessment tools for affective sound stimuli. 

The linear (two-tailed) Pearson correlation coefficients between the valence and arousal ratings obtained with the EmojiGrid (present study) and with the SAM ( Aguado *et al.,* 2018) were respectively 0.963 and 0.624 (p<0.001). To further quantify the agreement between both rating tools we computed intraclass correlation coefficients (ICC) with their 95% confidence intervals for the mean valence and arousal ratings between both studies. The ICC value for valence is 0.981 [0.967 – 0.989] while the ICC for arousal is 0.721 [0.509 – 0.842], indicating both studies show an excellent agreement for valence and a good agreement for arousal.

**Figure 5.**
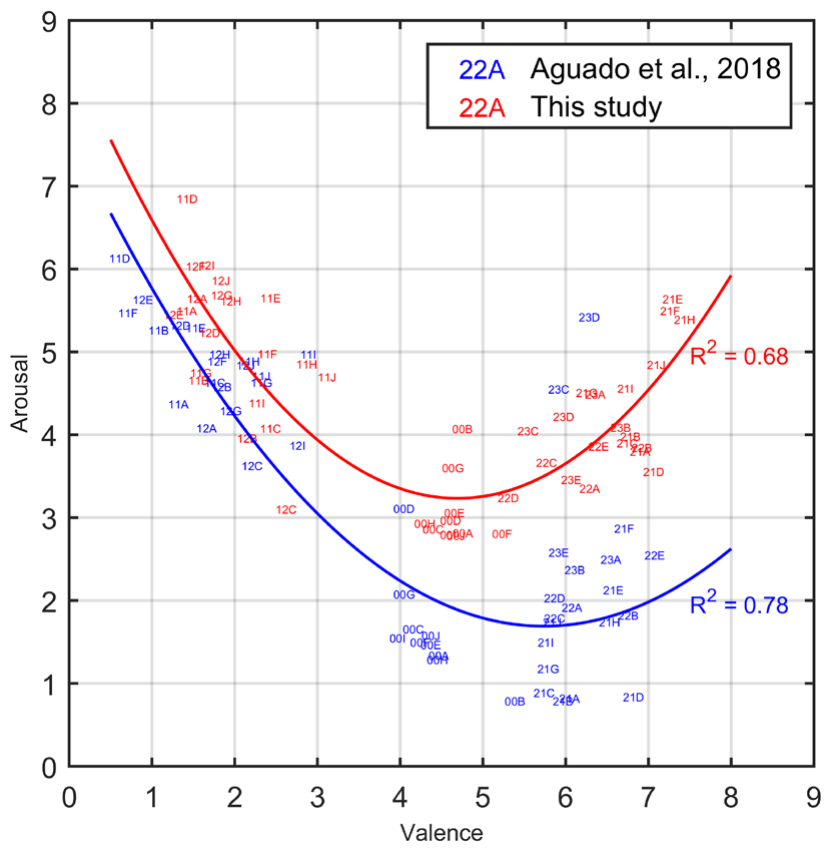
Mean valence and arousal ratings for affective film clips. Labels correspond to the original identifiers of the stimuli ( Aguado *et al.*, 2018). Blue labels represent data obtained with the SAM ( Aguado *et al.*, 2018) while red labels represent data obtained with the EmojiGrid (this study). The curves show quadratic fits to the corresponding data points.

Figure 5 shows the relation between the mean valence and arousal ratings for the 50 video clips tested. The curves in this figure represent quadratic fits to the data points. The adjusted R-squared values are respectively 0.68 and 0.78. Hence, both methods yield a relation between mean valence and arousal ratings that can be described by a quadratic (U-shaped) relation at the nomothetic (group) level.

Raw data from each experiment are available as *Underlying data* ( Toet, 2020).

## Conclusion

In this study we evaluated the recently developed EmojiGrid self-report tool for the affective rating of sounds and video. In two experiments, observers rated their affective appraisal of sound and video clips using the EmojiGrid. The results show a close correspondence between the mean ratings obtained with the EmojiGrid and those obtained with the validated SAM tool in previous validation studies in the literature: the agreement is excellent for valence and good for arousal, both for sound and video. Also, for both sound and video, the EmojiGrid yields the universal U-shaped (quadratic) relation between mean valence and arousal that is typically observed for affective sensory stimuli. We conclude that the EmojiGrid is an efficient affective self-report tool for the assessment of sound and video-evoked emotions.

A limitation of the EmojiGrid is the fact that it is based on the circumplex model of affect which posits that positive and negative feelings are mutually exclusive ( Russell, 1980). Hence, in its present form, and similar to other affective self-report tools like the SAM or VAS scales, the EmojiGrid only allows the measurement of a single emotion at a time. However, emotions are not strictly bipolar and two or more same or opposite valenced emotions can co-occur together ( Larsen & McGraw, 2014; Larsen *et al.*, 2001). Mixed emotions consisting of opposite feelings can in principle be registered with the EmojiGrid by allowing participants to enter multiple responses. 

Another limitation of this study is the fact that the comparison of the SAM and EmojiGrid ratings were based on ratings from different populations (akin to a comparison of two independent samples). Hence, our current regression estimates are optimized based on the particular samples that were used. Future studies should investigate a design in which the same participants use both self-report tools to rate the same set of stimuli.

Future applications of the EmojiGrid may involve the real-time evaluation of affective events or the provision of affective feedback. For instance, in studies on affective communication in human-computer interaction (e.g., Tajadura-Jiménez & Västfjäll, 2008), the EmojiGrid can be deployed as a continuous response tool by moving a mouse-controlled cursor over the grid while logging the cursor coordinates. Such an implementation may also afford the affective annotation of multimedia ( Chen *et al.,* 2007; Runge *et al.,* 2016), and could be useful for personalized affective video retrieval or recommender systems ( Hanjalic & Xu, 2005; Koelstra *et al.,* 2012; Lopatovska & Arapakis, 2011; Xu *et al.,* 2008), for real-time affective appraisal of entertainment ( Fleureau *et al.,* 2012) or to provide affective input to serious gaming applications ( Anolli *et al.,* 2010) and affective music generation ( Kim & André, 2004). Sensiks ( www.sensiks.com) has adopted a simplified version of the EmojiGrid in its Sensory Reality Pod to enable the user to select and tune multisensory (visual, auditory, tactile and olfactory) affective experiences.

## Data availability

### Underlying data

Open Science Framework: Affective rating of audio and video clips using the EmojiGrid. https://doi.org/10.17605/OSF.IO/GTZH4 ( Toet, 2020).

File ‘Results_sound_video’ (XLSX) contains the EmojiGrid co-ordinates selected by each participant following each stimulus.

Open Science Framework: Additional data on affective rating of audio and video clips using the EmojiGrid. https://doi.org/10.17605/OSF.IO/6HQTR

File ‘sound_results.xlsx’ contains the mean valence and arousal ratings, obtained with the SAM ( Yang *et al.,* 2018) and the EmojiGrid (this study), together with graphs in which each of the stimuli are labelled for easy identification.

File ‘video_results.xlsx’ contains the mean valence and arousal ratings, obtained with the SAM ( Aguado *et al.,* 2018) and the EmojiGrid (this study), together with graphs in which each of the stimuli are labelled for easy identification.

Data are available under the terms of the Creative Commons Attribution 4.0 International license (CC-BY 4.0).
