# Supplementary material for: Affective rating of audio and video clips using the EmojiGrid
Source: F1000Res. 2021 Apr 6;9:970. Originally published 2020 Aug 11. [Version 2] doi: 10.12688/f1000research.25088.2 (PMC8080979; doi:10.12688/f1000research.25088.2)
Supplement: Peer review history

# Peer review: Reviewer response for version 2

**DOI:** 10.5256/f1000research.55394.r82777

**Published:** 2021-04-23

**Authors:** Wei Ming Jonathan Phan

**Affiliations:** 1 California State University, Long Beach, Long Beach, CA, USA

Thank you very much the updated paper. The authors has addressed my concerns adequately.

Is the work clearly and accurately presented and does it cite the current literature?

Yes

If applicable, is the statistical analysis and its interpretation appropriate?

Partly

Are all the source data underlying the results available to ensure full reproducibility?

Yes

Is the study design appropriate and is the work technically sound?

Partly

Are the conclusions drawn adequately supported by the results?

Partly

Are sufficient details of methods and analysis provided to allow replication by others?

Partly

Reviewer Expertise:

Survey methodology, rating formats, Emotions, and Emojis.

I confirm that I have read this submission and believe that I have an appropriate level of expertise to confirm that it is of an acceptable scientific standard.

# Peer review: Reviewer response for version 2

**DOI:** 10.5256/f1000research.55394.r82776

**Published:** 2021-04-07

**Authors:** Linda K Kaye

**Affiliations:** 1 Department of Psychology, Edge Hill University, Ormskirk, UK

Thank you to the authors for making relevant changes as noted from the first round of reviews. I am satisfied that these have been addressed sufficiently.

Is the work clearly and accurately presented and does it cite the current literature?

Partly

If applicable, is the statistical analysis and its interpretation appropriate?

Yes

Are all the source data underlying the results available to ensure full reproducibility?

Yes

Is the study design appropriate and is the work technically sound?

Yes

Are the conclusions drawn adequately supported by the results?

Yes

Are sufficient details of methods and analysis provided to allow replication by others?

Partly

Reviewer Expertise:

Psychology of emoji; cyberpsychology; online behaviour

I confirm that I have read this submission and believe that I have an appropriate level of expertise to confirm that it is of an acceptable scientific standard.

# Peer review: Reviewer response for version 1

**DOI:** 10.5256/f1000research.27685.r76598

**Published:** 2021-01-11

**Authors:** Linda K Kaye

**Affiliations:** 1 Department of Psychology, Edge Hill University, Ormskirk, UK

This is an interesting study that seeks to validate the EmojiGrid for use with auditory and video stimuli. Thank you to the authors for providing the research resources on OSF as this is helpful when reviewing the research. Overall, the research has merits but would benefit from being more detailed especially in the introductory and discussion sections. I also have a methodological query but this may be rectified from additional clarity in the writing of this section.

1. The introduction could do with additional literature about the emotional affordances of emoji. That is, the research is presented as assuming that emoji are emotional stimuli but does not provide a review of the literature which can support this. Interestingly, recent evidence (Kaye *et al.*, 2021) suggests that emoji may not be processed emotionally on an implicit level, so the authors should be careful about their assumptions in this regard. Relevant sources that may be useful:
  Bai, Q., Dan, Q., Mu, Z., & Yang, M. (2019). A systematic review of emoji: Current research and future perspectives. *Frontiers in  Psychology, 10,* e2221.   doi:10.3389/fpsyg.2019.02221 ^1^
  Derks, D., Fischer, A. H., & Bos, A. E. R. (2008). The role of emotion in computer-mediated communication: A review. *Computers in Human Behavior, 24* (3), 766-785 ^2^
  Kaye, L. K., Rodriguez Cuadrado, S., Malone, S. A., Wall, H. J., Gaunt, E., Mulvey, A. L., & Graham, C. (2021). How emotional are emoji?: Exploring the effect of emotional valence on the processing of emoji stimuli. *Computers in Human Behavior, 116*, 106648 ^3^
  Novak, P. K., Smailović, J., Sluban, B., & Mozetič, I. (2015). Sentiment of emojis. *PLoS ONE*, *10* (12), e0144296 ^4^
2. With regards to the data presented (e.g., Fig 2), it is not made explicitly clear how numerical values were determined based on the responses from the EmojiGrid. E.g., how are each of the emoji symbols based on their position on the axis determined numerically? From Fig 1, it looks like this ranges from 1 to 5 based on the number of emoji on each axis. However, looking in the methodology, the SAM scale is outlined as being a 9-item response scale so it isn’t clear how Fig 2 & 3 can present the data from these two scales on the same axis if the response scales are different.
3. The discussion could benefit from further elaboration. E.g., To what extent do the findings contribute theoretically to the literature? What are the limitations of the work?

**Minor**

1. In the methodology, it is more typical to use the term “participants” rather than “persons”

Is the work clearly and accurately presented and does it cite the current literature?

Partly

If applicable, is the statistical analysis and its interpretation appropriate?

Yes

Are all the source data underlying the results available to ensure full reproducibility?

Yes

Is the study design appropriate and is the work technically sound?

Yes

Are the conclusions drawn adequately supported by the results?

Yes

Are sufficient details of methods and analysis provided to allow replication by others?

Partly

Reviewer Expertise:

Psychology of emoji; cyberpsychology; online behaviour

I confirm that I have read this submission and believe that I have an appropriate level of expertise to confirm that it is of an acceptable scientific standard, however I have significant reservations, as outlined above.

## References

[1] : A Systematic Review of Emoji: Current Research and Future Perspectives. *Frontiers in Psychology*.2019;10: 10.3389/fpsyg.2019.02221 10.3389/fpsyg.2019.02221 31681068PMC6803511

[2] : The role of emotion in computer-mediated communication: A review. *Computers in Human Behavior*.2008;24(3) : 10.1016/j.chb.2007.04.004 766-785 10.1016/j.chb.2007.04.004

[3] : How emotional are emoji?: Exploring the effect of emotional valence on the processing of emoji stimuli. *Computers in Human Behavior*.2021;116: 10.1016/j.chb.2020.106648 10.1016/j.chb.2020.106648

[4] : Sentiment of Emojis. *PLoS One*.2015;10(12) : 10.1371/journal.pone.0144296 e0144296 10.1371/journal.pone.0144296 26641093PMC4671607

# Response

**Published:** 2021-03-10

**Authors:** Alexander Toet

**Affiliations:** TNO, The Netherlands

Dear Dr Kaye,

 Thank you for your critical remarks and valuable suggestions which definitely helped us to improve our initial draft paper. Also, we appreciate the fact that you spent your valuable time on this review.

**1.  Literature about the emotional affordances of emoji**

 Thank you for this suggestion. We agree that reviewing literature about the emotional affordances of emoji will be a valuable addition to the Introduction, helping the reader to better place the current findings in their context. We therefore added the following text to the Introduction:

 “Emoji are facial icons that can elicit the same range of neural (Gantiva, Sotaquirá, Araujo, & Cuervo, 2020) and emotional (Moore, Steiner, & Conlan, 2013) responses as real human faces. In contrast to photographs, emoji are not associated with overgeneralization (the misattribution of emotions and traits to neutral human faces that merely bear a subtle structural resemblance to emotional expressions: Said, Sebe, & Todorov, 2009), or racial, cultural and sexual biases. Although some facial emoji can be poly-interpretable (Miller et al., 2016; Tigwell & Flatla, 2016) it has been found that emoji with similar facial expressions are typically attributed similar meanings (Jaeger & Ares, 2017; Moore et al., 2013) that are also to a large extent language independent (Kralj Novak, Smailović, Sluban, & Mozetič, 2015). Emoji have a wide range of different applications, amongst others in psychological research (Bai, Dan, Mu, & Yang, 2019). Emoji based rating tools are increasingly becoming popular tools as self-report instruments (Kaye, Malone, & Wall, 2017) to measure for instance user and consumer experience (e.g. www.emojiscore.com). Since facial expressions can communicate a wide variety of both basic and complex emotions emoji-based self-report tools may also afford the measurement and expression of mixed (complex) emotions that are otherwise hard to verbalize (Elder, 2018). However, while facial images and emoji are processed in a largely equivalent manner, suggesting that some non-verbal aspects of emoji are processed automatically, further research is required to establish whether they are also emotionally appraised on an implicit level (Kaye et al., 2021).”

**2.  How numerical values were determined**

 Thank you for pointing out this omission. We now include the following explanation of the scaling in the section on data analysis:

 “The response data (i.e., the horizontal or valence and vertical or arousal coordinates of the check marks on the EmojiGrid) were quantified as integers between 0 and 550 (the size of the square EmojiGrid in pixels), and then scaled between 1 and 9 for comparison with the results of  Yang et al. (2018) obtained with a 9-point SAM scale (Experiment I), or between 0 and 8 for comparison with the results of Aguado et al. (2018), also obtained with a  9-point SAM scale (Experiment II).”

**3.  Contribution and limitations**

 We now address some limitations of the present study (e.g. related to the measurement of mixed emotions and the study design itself) in the Discussion section (see also our reply to the comments of dr Pham).

**4. Minor points**

 We replaced “persons” by “participants” throughout the text.

# Peer review: Reviewer response for version 1

**DOI:** 10.5256/f1000research.27685.r69208

**Published:** 2020-09-01

**Authors:** Wei Ming Jonathan Phan

**Affiliations:** 1 California State University, Long Beach, Long Beach, CA, USA

Thank you for the opportunity to review the manuscript: “Affective rating of audio and video clips using the EmojiGrid.” This paper is primarily focused on validating the extension of a scale format (EmojiGrid) to a broader range of stimuli (audio and video). Overall, the paper makes some useful methodological contributions such as (1) the potentially greater ease for respondents for rating their emotions; (2) capturing both arousal and valence simultaneously; and (3) the use of more familiar contemporary symbols (emojis) compared to the SAM (Bradley & Lang, 1994). ^1^ I do have a few suggestions and concerns regarding the paper.

**1. Limitation of the EmojiGrid in measuring single discrete emotions.**

 The EmojiGrid is useful for respondents when selecting which area of the grid corresponds to their current felt emotion. However, emotions are not bipolar in nature and can often co-occur together, e.g., feeling bitter-sweet (Larsen *et al.*, 2001; Larsen & McGraw, 2014) ^2^^,^^3^. Thus, the current form of the EmojiGrid is limited to assessing stimuli that invoke single discrete emotions and may not be as suited for assessing real-time affective reactions (e.g., to entertainment or news). This limitation can potentially be highlighted in the discussion. Importantly, this limitation can be solved by future and different operationalizations of the grid structure when mixed emotions are the object of inquiry.

**2. Details regarding the stimuli selected.**

 Related to the first point, I note that the majority of the stimuli in both experiments (in particular experiment 1) seem to have a moderate amount of valence and arousal. Without knowing which stimuli were used, it is difficult to assess whether the emotion felt by the respondent was truly neutral or potential mix of emotions. To help the reader, please include two things potentially using tables in the supplementary material if needed. First, a greater description of which stimuli selected was expected to invoke which emotion in terms of both valence and arousal for both experiments. Second, please use a different numbering/labeling/coloring scheme that corresponds to the stimuli instead of dots for figures 1 and 2 when comparing the results from this study to previous work. Both are important because it allows the reader to visually assess the extent an expected emotion of stimuli (e.g., high arousal and positive valence) truly maps onto the mean scores and for the potential discrepancy between the two scale formats for the same stimuli to be obvious. This is important for replication but also because there is a greater dispersion when the SAM rating format is used.    

**3. Comparing current data and alternate (future) research design.**

 When comparing data from the current experiments to previous experiments the regression estimates are locally optimized based on the sample used to generate them. Thus, a caveat and clarification to potentially include are that the comparisons made are akin to that of two independent samples. Relatedly, an alternate design to consider would be doing a 4-block repeated measures design. Where participants rate the same stimuli using the two rating formats twice as:

 1. A then A

 2. B then B

 3. A then B

 4. B then A

 Blocks 3 and 4 would allow more direct comparisons between two different rating formats, especially given the greater dispersion in ratings observed when the SAM format is used.  

**4. Free response clicks within the EmojiGrid**

 I note that participants are free to click anywhere within the space of the EmojiGrid. I am curious as to variability/freedom that having no fixed anchor points generates. When participants respond do they more typically engage in: (1) subconsciously select a point close to one of the 25 potential points implied by the 5 X 5 grid of emojis, or (2) freely select a space with the grid, e.g., selecting point that corresponds to 2.30 arousal and 5.80 in valence? I ask this because the reliability of a scale is linked to the number of response points available (Preston & Colman, 2000; Schutz & Rucker, 1975) ^4^^,^^5^. If respondents are truly giving their ratings as (2) then greater reliability would be a potential additional advantage of using the EmojiGrid. If it were (1) the design of the EmojiGrid could include finer lines (i.e., more grid lines) to help respondents more easily locate their emotions on the Grid.

**Minor points**

1. Nationality information was collected from participants how was this information used? What was the distribution of nationalities for the participants?
2. I appreciate the way the authors determined their sample sizes.  

I enjoyed reading your paper and hope you will find my comments helpful!

Is the work clearly and accurately presented and does it cite the current literature?

Yes

If applicable, is the statistical analysis and its interpretation appropriate?

Partly

Are all the source data underlying the results available to ensure full reproducibility?

Yes

Is the study design appropriate and is the work technically sound?

Partly

Are the conclusions drawn adequately supported by the results?

Partly

Are sufficient details of methods and analysis provided to allow replication by others?

Partly

Reviewer Expertise:

Survey methodology, rating formats, Emotions, and Emojis.

I confirm that I have read this submission and believe that I have an appropriate level of expertise to confirm that it is of an acceptable scientific standard, however I have significant reservations, as outlined above.

## References

[1] : Measuring emotion: The self-assessment manikin and the semantic differential. *Journal of Behavior Therapy and Experimental Psychiatry*.1994;25(1) : 10.1016/0005-7916(94)90063-9 49-59 10.1016/0005-7916(94)90063-9 7962581

[2] : Can people feel happy and sad at the same time?. *Journal of Personality and Social Psychology*.2001;81(4) : 10.1037/0022-3514.81.4.684 684-696 10.1037/0022-3514.81.4.684 11642354

[3] : The Case for Mixed Emotions. *Social and Personality Psychology Compass*.2014;8(6) : 10.1111/spc3.12108 263-274 10.1111/spc3.12108

[4] : Optimal number of response categories in rating scales: reliability, validity, discriminating power, and respondent preferences. *Acta Psychologica*.2000;104(1) : 10.1016/S0001-6918(99)00050-5 1-15 10.1016/S0001-6918(99)00050-5 10769936

[5] : A Comparison of Variable Configurations Across Scale Lengths: An Empirical Study'. *Educational and Psychological Measurement*.1975;35(2) : 10.1177/001316447503500210 319-324 10.1177/001316447503500210

# Response

**Published:** 2021-03-10

**Authors:** Alexander Toet

**Affiliations:** TNO, The Netherlands

Dear Dr Phan,

 Thank you for your helpful suggestions and constructive remarks, which helped us to improve the quality of our initial draft paper. In addition, we appreciate the fact that you spent your valuable time on this review.

**1.         Mixed emotions**

 We thank the reviewer for raising this important issue. We now address this limitation in the Conclusion section as follows:

 “A limitation of the EmojiGrid is the fact that it is based on the circumplex model of affect which posits that positive and negative feelings are mutually exclusive (Russell, 1980).  Hence, in its present form, and similar to other affective self-report tools like the SAM or VAS scales, the EmojiGrid only allows the measurement of a single emotion at a time. However, emotions are not strictly bipolar and two or more same or opposite valenced emotions can co-occur together  (Larsen & McGraw, 2014; Larsen, McGraw, & Cacioppo, 2001). Mixed emotions consisting of opposite feelings can in principle be registered with the EmojiGrid by allowing participants to enter multiple responses. “

**2.  Stimulus details**

 Thank you for bringing this limitation to our attention. We agree that a labelling scheme (e.g. using the original stimuli identifier) makes a visual comparison between the experiments much easier. We therefore replaced the original graph with labelled graphs in the paper to allows the readers to visually assess and verify the expected emotions induced by the stimuli. We also added correlation plots for the mean valence and arousal ratings obtained both with the SAM and EmojiGrid to enable a direct comparison within each dimension.

 In addition, we now also provide a more detailed description of the selected stimuli in a new set of Excel notebooks that we uploaded to the Open Science Framework  These notebooks include a brief description of the nature and content of all stimuli, their original affective classification, and their mean valence and arousal values (1) as provided by the authors of the (sound and video) databases and (2) as measured in this study.  

 The notebooks also contain several graphs in which each of the stimuli is represented by the index number for easy identification. The graphs include plots showing (1) the relation between the mean valence measures obtained with the SAM and EmojiGrid, (2) the  relation between the mean arousal measures obtained with the SAM and EmojiGrid, (3) the relation between the mean valence and arousal measures obtained with the SAM, and (4) the relation between the mean valence and arousal measures obtained with the EmojiGrid.

**3. Comparing data**

 Thank you for pointing out this limitation.  We now address this issue in the Conclusion section as follows:

 “Another limitation of this study is the fact that the comparison of the SAM and EmojiGrid ratings were based on ratings from different populations (akin to a comparison of two independent samples). Hence, our current regression estimates are optimized based on the particular samples that were used. Future studies should investigate a design in which the same participants use both self-report tools to rate the same set of stimuli. “

**4. Free response clicks**

 Thank you for raising this potentially important issue. We plotted the raw response data for visual inspection. The overall response pattern appears truly random and shows no regularities or evidence for attraction to any of the individual emojis lining the grid area or to any of the grid lines inside the grid area.

**Minor points**

 Thank you for drawing our attention to this omission. All participants had the UK nationality. We now report this in the text. Nationality information was collected to check if the participants adhered to the recruitment restrictions as specified through Prolific.

 Thank you for your positive appraisal. Your comments were quite valuable, and definitely served to improve the quality of our paper.
